# Impact of parental divorce versus separation due to migration on mental health and self-injury of Chinese children: a cross sectional survey

**DOI:** 10.1186/s13034-021-00424-z

**Published:** 2021-11-27

**Authors:** Feng Wang, Jingjing Lu, Leesa Lin, Jingjing Cai, Jiayao Xu, Xudong Zhou

**Affiliations:** 1 The Institute of Social and Family Medicine, School of Medicine, Zhejiang University, Hangzhou, Zhejiang People’s Republic of China; 2 Department of Infectious Disease Epidemiology, London School of Hygiene & Tropical Medicine, Kings Cross, London, UK

**Keywords:** Parental divorce, Left-behind children, Mental health, Parent-adolescent communication, Psychological resilience

## Abstract

**Background:**

There has been an increasing prevalence of parental separation in China due to divorce or migration for work in recent decades. However, few studies have compared the impacts of these two types of separation on children’s mental health. This study aimed to investigate how parental divorce and parental migration impact children’s mental health and self-injurious thoughts and behaviors (SITB), while considering positive factors, including parent-adolescent communication and psychological resilience.

**Methods:**

We randomly recruited participants in grades 5–8 from 18 schools in 2 counties in Anhui Province. A self-administered questionnaire was conducted to measure children’s mental health, SITB, parent-adolescent communication, psychological resilience, and socio-demographic characteristics.

**Results:**

Data from 1026 children with both parents migrating (BLBC), 1322 children with one parent migrating (SLBC), 475 children living in a divorced family (DC) and 1160 children with non-migrating parents (NLBC) were included. Regression model results showed that, compared to the other three groups (BLBC, SLBC, NLBC), DC exhibited higher internalizing problems (p < 0.05), higher externalizing problems (p < 0.01), less prosocial behaviors (p < 0.05), and higher rates of suicidal ideation (SI) (p < 0.05) and non-suicidal self-injury (NSSI) behaviors (p < 0.05) when adjusting for social-demographic variables. However, when further adjusting for parent-adolescent communication and psychological resilience, DC no longer had higher levels of internalizing problems, externalizing problems, NSSI and SI than left-behind groups (BLBC, SLBC).

**Conclusions:**

The experience of separation from divorced parents had stronger negative effects on the mental health of children than was observed in LBC. The Chinese government should design special policy frameworks that provide support to DC.

## Background

Parental absence, due to divorce or migration, has been regarded as the most common type of childhood adversity [1]. A number of studies suggest that adverse childhood experiences are a major risk factor for the development of mental health disorders [2]. In light of these facts, two demographic trends that have emerged in China within the last decade deserve greater attention. Firstly, with the changes in people’s ideation, women’s education and economic status [3], urbanization [4] and internal migration [5], China’s crude divorce rate has dramatically increased from 0.85 per 1000 persons in 1979 to 3.26 per 1000 persons in 2018 [6]. Because of this, a large number of children are now at increased risk of experiencing the detrimental consequences associated with parental divorce.

Secondly, China has experienced a massive population exodus from rural to urban areas, spurred by the acceleration of urbanization and industrialization in China during the past four decades. The Chinese migrant population is estimated to be 245 million, and accounts for roughly 30% of the working population in China [7]. Because of stringent entry policies, financial constraints, and limited access to public goods in their destination cities, many migrants leave their children behind in their hometown while migrating [8]. This has resulted in an estimated 68.7 million children aged 18 years or younger being left behind by their parents in their home communities, who were known as left-behind children (LBC) [9].

Therefore, it is not surprising that increasing divorce rates and parental separation due to migration have raised public concerns about potential negative effects on children. Research on children whose parents have divorced has documented significant short- and long-term consequences associated with childhood separation from parents, including depression [10–12], suicide attempts [13], suicidal ideation [14], and higher risk of substance use behaviors [15, 16].

Many researchers have also sought to determine the effects of parental migration on children in the past decade. Previous studies have found that parental migration is a factor which is strongly related to internalizing problems [17–19], externalizing problems [20–22], low self-esteem [23], and suicidal ideation [24–26]. However, the risk for psychopathology associated with separation from parents appears to vary by the form of separation. For example, children who experienced the divorce, but not the death of a parent, appear to have higher levels of depression and anxiety [27]. This finding that parental divorce had a stronger effect than did a parental death, has been buttressed by further research [28, 29]. Despite the frequency of parental divorce and the severity of parental migration, few studies have differentiated between the psychological consequences of parental divorce and parental migration.

As research on parental separation and children’s adaptation to separation has accumulated, there has been a gradual shift in emphasis from family structure to family process and protective factors, such as parent-adolescent communication and psychological resilience [30]. Previous research indicated that high levels of parent-adolescent communication and psychological resilience can protect the mental health of children and reduce their risk of suicide attempts. As an indicator of the strength of parent–child relationship, parent-adolescent communication is emphasized as an important family factor contributing to children’s development [31]. For instance, a study on 2707 children in Belgium demonstrated that experiencing problems in communicating with parents was significantly related to children’s self-harm behavior and suicidal ideation [32]. Our previous studies also showed that LBC in China have lower quality of parent-adolescent communication than do their non-left-behind counterparts, and that experiencing problems in communicating with their parents was associated with higher levels of mental health difficulties [19, 33]. Moreover, there is well-established evidence from the fields of positive psychology that psychological resilience can moderate the effects of adverse life events on adolescent mental health disorders [34–36]. However, previously collected data were limited concerning protective factors for children, as differentiated by patterns of parental separation in China.

The aim of this study was to investigate the effects of diverse forms of parental absence on the mental health and self-injurious thoughts and behaviors (SITB) of children in China, including children left behind by both parents (BLBC), those left behind by one parent (SLBC) and those with divorced parents (DC). We were also interested in how parent-adolescent communication and psychological resilience affects children with respect to different forms of parental separation status.

## Methods

### Participants and survey procedure

We conducted a cross-sectional survey in Anhui province in southeast China from April 2018 to March 2019. As a relatively underdeveloped province which is the source of many migrants, Anhui houses nearly 16 million migrant workers and around 4.5 million LBC [37]. As Wuwei and Nanling counties report the large numbers of LBC in Anhui province, they were selected as the study sites. Within each county, one urban district and two rural towns were randomly selected. Then, two random schools in each selected district/township were included in the survey. In each school, all students from grades 5–6 (primary school) and grades 7–8 (middle school) were invited to participate. The current study specifically focused on early adolescence (grade 5 to grade 8), as this age period is generally perceived as the starting point of the dramatic physical and cognitive changes associated with puberty is associated with changes in social status, and is a period of developmental opportunity and vulnerability [38].

The required sample size is 3500, which was calculated in our previous study [33]. Prior to the survey, written consent was obtained from eligible participants and their parents/guardians. All students were informed of the purpose and procedures of the survey in detail. Those who agreed to participate were asked to complete a self-reported questionnaire in their classroom. To avoid potential contamination, questionnaires were completed under exam conditions and discussions between students were not allowed. A group of research assistants was trained and present in the classroom to clarify any potential confusing items and answer questions. No one except the researchers received access to the information written in the questionnaire. Anonymity and confidentiality were assured. The Ethical Committee of the School of Public Health of Zhejiang University approved this study.

### Measures

#### Parent-adolescent communication

The Chinese version of the parent-adolescent communication scale (PACS) was used to assess communication between students and their parents [39, 40]. This 20-item scale is divided into two sub-scales: communication openness with parents (10 items) and communication problems with parents (10 items). Greater values in the openness subscale scores indicate a more open and healthy level of parent–child communication, while greater values in the problem subscale scores indicates a higher degree of problems. These two subscales were taken together to calculate the total scale score. Higher scores on the total scale represent better parent-adolescent communication. Both the original version and Chinese version of this scale have an acceptable level of reliability [39, 41]. The Cronbach’s α for the openness subscale, problems subscale and the total scale in the current study sample were 0.93, 0.83 and 0.87 for fathers, and 0.90, 0.79 and 0.84 for mothers, respectively.

#### Psychological resilience

Psychological resilience was measured with the Chinese version of the Connor-Davidson Resilience Scale (CD-RISC) [42, 43]. This scale contains 25 items, such as “able to adapt to change,” and “tend to bounce back after illness or hardship.” Participants were asked to respond on how they felt during the previous month. A 5-point Likert scale was used, from 0 (not true at all) to 4 (true nearly all of the time). Total scores range from 0 to 100, with higher scores indicating greater resilience. This scale has demonstrated high levels of internal consistency (Cronbach’s α = 0.91) and test–retest reliability (intra-class correlation coefficient = 0.87) among Chinese samples [44]. In the present study, Cronbach’s α for the CD-RISC was 0.92.

#### Mental health

Children’s mental health was measured with the self-reported version of the Strengths and Difficulties Questionnaire (SDQ), which has been validated in China [45, 46]. The SDQ is a brief but comprehensive screening tool for child and adolescent mental health and has become one of the most widely used measurement tools globally [47]. The SDQ has four subscales to measure difficulties (emotional symptoms, conduct problems, hyperactivity, and peer problems) and one further subscale to assess strengths (pro-social behaviors). Each subscale comprises five items: each item is scored from 0 to 2. Emotional symptoms and peer problems were combined into a single “internalizing” subscale, conduct problems and hyperactivity were combined to form a single “externalizing” subscale, and the third subscale, “pro-social behavior,” remained unchanged [48]. Total difficulties scores were obtained by adding the scores of the internalizing and externalizing subscales. Regarding the total difficulties and internalizing and externalizing subscales, higher scores indicate higher levels of difficulties. In the pro-social subscale, however, higher scores represent higher levels of strength. The Cronbach’s α for emotional symptoms in the current study was 0.74; 0.78 for conduct problems; 0.72 for hyperactivity; 0.67 for peer problem; and 0.76 for the pro-social behavior.

#### Non-suicidal self-injury and suicidal ideation

Self-injurious thoughts and behaviors (SITB), including non-suicidal self-injury (NSSI), suicidal ideation (SI), suicidal plans and suicide attempts, are widely used to obtain information regarding adolescent suicidality [49]. We only assessed the NSSI and SI in this study. The presence of NSSI and SI were assessed by using two questions based on the Composite International Diagnostic Interview (CIDI) [50] referring to the past 12 months: “Did you hurt yourself deliberately without the intent of suicide? (any form of those behaviors: hitting, pulling hair, head banging, pinching, scratching, biting, burning and cutting)” and “Did you ever have suicidal thoughts?” The following statements were identified as a “yes” answer for NSSI: “During the past year, I have hurt myself deliberately more than once.” Participants were categorized into four groups based on their answers about their SI, including: “I do not have any thoughts of killing myself”, “I have thoughts of killing myself but I wouldn’t carry them out”, “I would like to kill myself” and “I would kill myself if I had the chance”, respectively.

These questions have demonstrated substantial reliability. The two-week test–retest was 0.84 for NSSI, and 0.79 for SI [51].

#### Socio-demographic variables

Socio-demographic characteristics collected for this study included: age, gender (male/female), grade (grade 5–6/grade 7–8), perceived family income level (much better off/better off/the same/poorer/much poorer), parental highest education level (primary school or lower/middle school/high school or above/do not know), parents’ marriage status (divorced/non-divorced) and household registration (rural/urban).

#### Parental absence status

We applied a strict inclusion and exclusion criteria to identify participants for the current study: (1) for BLBC, both father and mother were currently migrating to other places for work and were absent for over six months (non-divorced family); for SLBC, one parent was currently migrating away for work and no longer lived with their children for a period of more than 6 months (non-divorced family); for NLBC, both parents lived in the household and neither had ever migrated elsewhere for work (non-divorced family); (2) for DC, living in a divorced family without migrant parents. Considering that different forms of parental absence could exert differing effects on children, the exclusion criteria included: (1) children whose parents have passed away; and (2) living in a step-parent family. Based on these criteria, we identified 1026 BLBC, 1322 SLBC, 475 DC and 1160 NLBC in the final study sample. Of the total sample, 52 (1.3%) declined to answer the questionnaire, and a further 76 (1.9%) failed to report their parental separation status.

### Statistical analysis

Analyses of Variance (ANOVA, for continuous variables) or chi-square tests (for categorical variables) were conducted to compare sample characteristics, PACS, CD-RISC, SDQ, NSSI and SI among the four groups of children with different parental separation status. Multiple linear regression models were applied to examine the associations between the SDQ outcomes and parental absence status. Binary logistic regression models were performed to explore the effects of different forms of parental separation on children’s NSSI and SI. The initial model was adjusted for sample demographics (age, gender, income level, parental highest education level, sibling and household registration). The model was further adjusted for parent-adolescent communication and psychological resilience. The significance level was set at 0.05, and all the tests were two-sided. Data management and all analyses were performed using SPSS 24.0 version (IBM Corp., NY, USA).

## Results

Table 1 presents the descriptive statistics of children by their parental separation status. Mean age of the sample was 13.0 (SD 1.3) with NLBC being slighter younger (mean 12.8, SD 1.3) than the other three groups. Overall, there were more boys (55.4%) than girls (44.6%) in the study sample and the gender distribution did not differ across the four groups. Children whose parents had divorced were more likely to live in economically disadvantaged situations. About one-sixth of DC reported that they were from poorer households, whereas the percentages for BLBC, SLBC and NLBC were only 6.4, 9.9 and 6.0%, respectively. Approximately 10% of the participants in this study did not know their parents’ education level, while NLBC’s parents demonstrated the highest level of education. The proportions of one-child families were highest for the DC (55.9%), and lowest for the SLBC group (29.1%). When compared to left-behind groups, NLBC were more likely to live in urban areas (76.9%).

**Table 1:** Demographic characteristics of study participants, n (%)

	BLBC n = 1026	SLBC n = 1322	DC n = 475	NLBC n = 1160	F or χ^2^	*P* value
Age, mean (SD)	13.1 (1.3)	13.1 (1.3)	13.0 (1.3)	12.8 (1.3)	14.71	< 0.001
Gender					4.00	0.261
Male	569 (56.2)	760 (58.3)	248 (53.8)	630 (55.1)		
Female	443 (43.8)	543 (41.7)	213 (46.2)	513 (44.9)		
Grade					17.30	0.001
Grade 5 Grade 6	449 (43.8)	547 (41.4)	219 (46.1)	574 (49.5)		
Grade 7 Grade 8	576 (56.2)	774 (58.6)	256 (53.9)	585 (50.5)		
Income level					72.10	< 0.001
Much better off/better off	280 (27.6)	315 (24.0)	80 (17.0)	341 (29.9)		
The same	670 (66.0)	865 (66.0)	315 (67.0)	731 (64.1)		
Poorer/much poorer	65 (6.4)	130 (9.9)	75 (16.0)	68 (6.0)		
Parental highest education level					98.58	< 0.001
Primary school or lower	126 (12.3)	194 (14.7)	65 (13.7)	116 (10.0)		
Middle school	597 (58.2)	723 (54.7)	229 (48.2)	519 (44.7)		
High school or above	190 (18.5)	292 (22.1)	119 (25.1)	388 (33.4)		
Do not know	113 (11.0)	113 (8.5)	62 (13.1)	137 (11.8)		
Only child					114.14	< 0.001
No	690 (67.3)	936 (70.9)	209 (44.1)	764 (65.9)		
Yes	335 (32.7)	385 (29.1)	265 (55.9)	395 (34.1)		
Household registration					455.30	< 0.001
Rural	699 (68.1)	552 (41.8)	232 (48.8)	268 (23.1)		
Urban	327 (31.9)	770 (58.2)	243 (51.2)	892 (76.9)		

*LBC* left-behind children, *BLBC* LBC with both parents migrating, *SLBC* LBC with one parent migrating, *NLBC* neither parents had migrated, *DC* children living in a divorced family without migrant parents

The observed differences in mean total and sub-scale scores from PACS and CD-RISC among the four groups of children are displayed in Table 2. Significant differences were found among different groups of students in regards to father-child openness score, father-child problem score, father-child total score, mother–child openness score, mother–child problem score, and mother–child total score. The DC had the lowest scores on the openness sub-scales and total scales of father- and mother-adolescent communication, and had the highest scores on the problem subscales of father- and mother-adolescent communication, as compared to the other groups. In respect to CD-RISC, DC reported the lowest scores on the resilience outcome (F = 21.84, p < 0.001).

**Table 2:** Parent–child communication and psychological resilience outcomes by parental absence status, mean (SD)

	BLBC	SLBC	DC	NLBC	F	P value
Mother-adolescent communication						
Openness subscale (10–50)^@^	29.4 (6.0)	29.0 (6.4)	27.6 (7.1)	30.0 (6.2)	17.13	< 0.001
Problem subscale (10–50)^#^	22.0(5.2)	22.6 (5.2)	22.8 (5.6)	21.4 (5.3)	12.19	< 0.001
Total scale (20–100)^$^	57.5 (10.2)	56.5 (10.5)	54.8 (11.0)	58.6 (10.4)	16.52	< 0.001
Father-adolescent communication						
Openness subscale (10–50)^%^	28.9 (6.6)	29.4 (6.7)	27.0 (7.1)	29.9 (6.7)	21.95	< 0.001
Problem subscale (10–50)^&^	21.2 (5.4)	20.5 (5.4)	22.3 (5.6)	19.9 (5.4)	25.39	< 0.001
Total scale (20–100)^*^	57.8 (10.8)	58.9 (11.0)	54.8 (11.5)	60.2 (11.1)	27.60	< 0.001
Resilience total score (0–100)^!^	57.5 (16.5)	58.6 (16.0)	53.8 (16.2)	60.9 (16.3)	21.84	< 0.001

*LBC* left-behind children, *BLBC* LBC with both parents migrating, *SLBC* LBC with one parent migrating; *NLBC* neither parents had migrated; *DC* children living in a divorced family without migrant parents

^@^: Post-hoc, (1,3), (2,3), (2,4), (3,4); #: Post-hoc, (2,4), (3,4); $: Post-hoc, (1,3), (2,3), (2,4), (3,4);

^%^: Post-hoc, (1,3), (1,4), (2,3), (3,4); &: Post-hoc, (1,2), (1,3), (1,4), (2,3), (2,4), (3,4);

*: Post-hoc, (1,3), (1,4), (2,3), (2,4), (3,4); !: Post-hoc, (1,3), (1,4), (2,3), (2,4), (3,4)

Table 3 illustrates the differences in mental health outcomes across the four groups of children. DC had significantly higher mean scores for total difficulties, internalizing problems and externalizing problems, and lower mean scores on the pro-social behavior sub-scale compared to the other groups. Table 3 also shows the percentages of individual NSSI and SI across different groups of participants. DC reported significantly higher rates of NSSI (18.1%, p = 0.002) and SI (22.3%, p = 0.002).

**Table 3:** Strength and Difficulties Questionnaire, non-suicidal self-injury and suicidal ideation: comparison of four groups, mean (SD)/n(%)

	BLBC	SLBC	DC	NLBC	F or χ^2^	P value
Emotional symptoms (0–10)^a^	3.6 (2.2)	3.4 (2.2)	4.0 (2.4)	3.1 (2.2)	21.11	< 0.001
Conduct problems (0–10)^b^	2.5 (1.6)	2.5 (1.7)	2.8 (1.8)	2.3 (1.6)	10.90	< 0.001
Hyperactivity (0–10)^c^	3.9 (2.2)	4.0 (2.2)	4.5 (2.3)	3.6 (2.2)	19.57	< 0.001
Peer problems (0–10)^d^	2.7 (1.7)	2.7 (1.7)	2.9 (1.8)	2.5 (1.7)	7.20	< 0.001
Total difficulties score (0–40)^e^	12.7 (5.5)	12.5 (5.6)	14.2 (6.0)	11.5 (5.5)	27.64	< 0.001
Pro-social (0–10)^f^	7.0 (2.0)	7.0 (2.1)	6.8 (2.1)	7.1 (2.0)	4.68	0.003
Internalizing problems (0–20)^g^	6.2 (3.2)	6.1 (3.2)	6.9 (3.4)	5.6 (3.1)	21.47	< 0.001
Externalizing problems (0–20)^h^	6.4 (3.3)	6.4 (3.3)	7.3 (3.5)	5.9 (3.4)	20.17	< 0.001
Non-suicidal self-injury^i^					15.05	0.002
Yes	158 (15.4)	181 (13.7)	86 (18.1)	132 (11.4)		
No	868 (84.6)	1141 (86.3)	389 (81.9)	1028 (88.6)		
Suicidal ideation^j^					26.17	0.002
I do not have any thoughts of killing myself	761 (74.2)	959 (72.7)	319 (67.2)	900 (77.7)		
I have thoughts of killing myself, but I would not carry them out	242 (23.6)	319 (24.2)	142 (29.9)	237 (20.4)		
I would like to kill myself	18 (1.8)	29 (2.2)	7 (1.5)	17 (1.5)		
I would kill myself if I had the chance	5 (0.5)	12 (0.9)	7 (1.5)	5 (0.4)		

*LBC* left-behind children, *BLBC* LBC with both parents migrating, *SLBC* LBC with one parent migrating, *NLBC* neither parents had migrated, *DC* children living in a divorced family without migrant parents

^a^: Post-hoc, (1,3), (1,4), (2,3), (2,4), (3,4); ^b^: Post-hoc, (1,3), (2,3), (2,4), (3,4)

^c^: Post-hoc, (1,3), (1,4), (2,3), (2,4), (3,4); ^d^: Post-hoc, (1,3), (2,3), (3,4); ^e^: Post-hoc, (1,3), (1,4), (2,3), (2,4), (3,4); f: Post-hoc, (3,4)

^g^: Post-hoc, (1,3), (1,4), (2,3), (2,4), (3,4); ^h^: Post-hoc, (1,3), (1,4), (2,3), (2,4), (3,4); ^i^: Post-hoc, (1,4), (3,4); ^j^: Post-hoc, (3,4)

Tables 4 and 5 display the multiple linear regression analyses of SDQ outcomes and the binary logistic regression analyses of SITB outcomes. When adjusting for socio-demographic variables, BLBC, SLBC and NLBC had lower total difficulties scores (β = − 1.19, 95%CI = − 1.88, − 0.50, p < 0.01; β = − 1.28, 95%CI = − 1.95, − 0.62, p < 0.001; β = − 2.20, 95%CI = − 2.88, − 1.51, p < 0.001), lower internalizing problems scores (β = − 0.48, 95%CI = − 0.87, − 0.09, p < 0.05; β = − 0.62, 95%CI = − 1.00, − 0.24, p < 0.01; β = − 1.04, 95%CI = − 1.43, − 0.65, p < 0.001) and lower externalizing problems scores (β = − 0.70, 95%CI = − 1.11, − 0.29, p < 0.01; β = − 0.66, 95%CI = − 1.06, − 0.26, p < 0.01; β = − 1.15, 95%CI = − 1.56, − 0.75, p < 0.001) than DC, but they scored higher on pro-social behavior (β = 0.27, 95%CI = 0.02, 0.52, p < 0.05; β = 0.32, 95%CI = 0.08, 0.56, p < 0.01; β = 0.31, 95%CI = 0.07, 0.56, p < 0.05). After adjusting for all PACS and CD− RISC variables, the decreases in total difficulties scores (β = − 0.21, 95%CI = − 0.80, 0.38, p = 0.481; β = − 0.59, 95%CI = − 1.16, 0.03, p = 0.226), internalizing problems scores (β = − 0.05, 95%CI = − 0.41, 0.31, p = 0.787; β = − 0.30, 95%CI = − 0.65, 0.05, p = 0.095), externalizing problems scores (β = − 0.15, 95%CI = − 0.50, 0.21, p = 0.415; β = − 0.27, 95%CI = − 0.62, 0.07, p = 0.119) and increases in pro-social behavior score (β = − 0.10, 95%CI = − 0.30, 0.11, p = 0.350; β = − 0.03, 95%CI = − 0.22, 0.17, p = 0.800) were no longer significant in BLBC and SLBC when compared to DC. Furthermore, BLBC, SLBC and NLBC were less likely to engage in NSSI and SI than DC, but only in the initial model.

**Table 4:** Regression analysis for mental health by parental absent groups with adjustment for socio-demographic characteristics, β(95%CI)

	Emotional symptoms^@^		Conduct problems^#^		Hyperactivity^$^	
Parental migration status (ref: DC)						
BLBC	−0.30 ( −0.57, −0.02)^*^	−0.01 ( −0.27, 0.24)	−0.20 ( −0.41, 0.01)	0.01 ( −0.19, 0.20)	−0.53 ( −0.79, −0.26)^***^	−0.17 ( −0.40, 0.06)
SLBC	−0.42 ( −0.69, −0.16)^**^	−0.24 ( −0.48, 0.01)	−0.22 ( −0.42, −0.02)^*^	−0.10 ( −0.28, 0.09)	−0.47 ( −0.72, −0.21)^***^	−0.20 ( −0.42, 0.03)
NLBC	−0.75 ( −1.02, −0.47)^***^	−0.47 ( −0.72, −0.22)^***^	−0.45 ( −0.66, −0.25)^***^	−0.27 ( −0.45, −0.08)^**^	−0.72 ( −0.99, −0.46)^***^	−0.33 ( −0.56, −0.10)^**^
Mother–adolescent communication						
Openness communication		0 ( −0.01, 0.02)		−0.02 ( −0.03, −0.01)^**^		−0.02 ( −0.03, −0.01)^*^
Problem communication		0.12 (0.10, 0.13)^***^		0.07 (0.06, 0.09)^***^		0.09 (0.07, 0.10)^***^
Father–adolescent communication						
Openness communication		−0.01 ( −0.03, 0.01)		0.01 (0, 0.02)		0 ( −0.01, 0.02)
Problem communication		0.05 (0.03, 0.07)^***^		0.05 (0.03,0.06)^***^		0.04 (0.02, 0.06)^***^
Resilience		−0.02 ( −0.02, −0.01)^***^		−0.01 ( −0.01, −0.01)^***^		−0.04 ( −0.05, −0.04)^***^

	Peer problems^%^		Total difficulties^&^		Pro-social^!^	
Parental migration status (ref: DC)						
BLBC	−0.17 ( −0.38, 0.03)	−0.02 ( −0.22, 0.18)	−1.19 ( −1.88, −0.50)^**^	−0.21 ( −0.80, 0.38)	0.27 (0.02, 0.52)^*^	−0.10 ( −0.30, 0.11)
SLBC	−0.18 ( −0.38, 0.02)	−0.03 ( −0.23, 0.16)	−1.28 ( −1.95, −0.62)^***^	−0.59 ( −1.16, 0.03)	0.32 (0.08, 0.56)^**^	−0.03 ( −0.22, 0.17)
NLBC	−0.27 ( −0.48, −0.07)^**^	−0.08 ( −0.28, 0.11)	−2.20 ( −2.88, −1.51)^***^	−1.18 ( −1.76, −0.60)^***^	0.31 (0.07, 0.56)^*^	−0.15 ( −0.35, 0.06)
Mother–adolescent communication						
Openness communication		−0.02 ( −0.03, −0.01)^**^		−0.06 ( −0.09, −0.02)^**^		0.05 (0.04, 0.06)^***^
Problem communication		0 ( −0.01, 0.02)		0.28 (0.23, 0.32)^***^		0.01 ( −0.01, 0.02)
Father–adolescent communication						
Openness communication		0 ( −0.01, 0.02)		0 ( −0.03, 0.04)		0.01 (0,0.02)
Problem communication		0.04 (0.02, 0.05)^***^		0.17 (0.13, 0.22)^***^		−0.01 ( −0.02, 0.01)
Resilience		−0.02 ( −0.02, −0.02)^***^		−0.09 ( −0.10, −0.08)^***^		0.06 (0.06, 0.07)^***^

*LBC* left-behind children, *BLBC* LBC with both parents migrating, *SLBC* LBC with one parent migrating, *NLBC* neither parents had migrated, *DC* children living in a divorced family without migrant parents

Adjusted by age, gender, income level, parental highest education level, sibling and household registration. *: p < 0.05; **: p < 0.01; ***: p < 0.001

^@^: n = 3899, adjusted R^2^ = 0.037, F = 12.98, p < 0.001; n = 3899, adjusted R^2^ = 0.191, F = 51.27, p < 0.001

^#^: n = 3891, adjusted R^2^ = 0.010, F = 4.14, p < 0.001; n = 3891, adjusted R^2^ = 0.156, F = 40.19, p < 0.001

^$^: n = 3899, adjusted R^2^ = 0.032, F = 11.24, p < 0.001; n = 3899, adjusted R^2^ = 0.273, F = 80.85, p < 0.001

^%^: n = 3896, adjusted R^2^ = 0.025, F = 8.89, p < 0.001; n = 3896, adjusted R^2^ = 0.10, F = 24.12, p < 0.001

^&^: n = 3851, adjusted R^2^ = 0.028, F = 9.89, p < 0.001; n = 3851, adjusted R^2^ = 0.309, F = 94.83, p < 0.001

!: n = 3897, adjusted R^2^ = 0.035, F = 12.18, p < 0.001; n = 3897, adjusted R^2^ = 0.357, F = 118.76, p < 0.001

**Table 5:** Regression analysis for internalizing and externalizing problems, non-suicidal self-injury and suicidal ideation by parental absent groups, β/OR (95% CI)

	Internalizing problems^@^		Externalizing problems^#^	
Parental migration status (ref: DC)				
BLBC	− 0.48 (− 0.87, − 0.09)^*^	− 0.05 (− 0.41, 0.31)	− 0.70 (− 1.11, − 0.29)^**^	− 0.15 (− 0.50, 0.21)
SLBC	− 0.62 (− 1.00, − 0.24)^**^	− 0.30 (− 0.65, 0.05)	− 0.66 (− 1.06, − 0.26)^**^	− 0.27 (− 0.62, 0.07)
NLBC	− 1.04 (− 1.43, − 0.65)^***^	− 0.59 (− 0.94, − 0.23)^**^	− 1.15 (− 1.56, − 0.75)^***^	− 0.58 (− 0.93,− 0.23)^**^
Mother-adolescent communication				
Openness communication		− 0.02 (− 0.04, 0)		− 0.04 (− 0.06,− 0.02)^**^
Problem communication		0.12 (0.09, 0.15)^***^		0.16 (0.13,0 .19)^***^
Father-adolescent communication				
Openness communication		− 0.01 (− 0.03, 0.02)		0.01 (− 0.01, 0.03)
Problem communication		0.09 (0.06, 0.12)^***^		0.09 (0.06, 0.11)^***^
Resilience		− 0.03 (− 0.04,− 0.03)^***^		− 0.05 (− 0.06,− 0.05)^***^

	Self-injury^$^		Suicidal ideation^%^	
Parental migration status (ref: DC)				
BLBC	0.76 (0.54, 0.98)^*^	0.95 (0.67, 1.34)	0.69 (0.52, 0.91)^**^	0.90 (0.67, 1.21)
SLBC	0.71 (0.52, 0.96)^*^	0.81 (0.58, 1.13)	0.76 (0.58, 0.98)^*^	0.90 (0.68, 1.19)
NLBC	0.60 (0.42, 0.84)^**^	0.72 (0.50, 1.02)	0.57 (0.43, 0.75)^***^	0.71 (0.53, 0.95)^*^
Mother-adolescent communication				
Openness communication		0.97 (0.95, 0.99)^*^		0.96 (0.95, 0.98)^***^
Problem communication		1.08 (1.05, 1.11)^***^		1.09 (1.07, 1.12)^***^
Father-adolescent communication				
Openness communication		0.99 (0.97, 1.01)		0.96 (0.95, 0.98)^***^
Problem communication		1.03 (1.00, 1.06)		1.01 (0.99, 1.03)
Resilience		0.99 (0.98, 0.99)^*^		0.99 (0.98, 0.99)^***^

*LBC* left-behind children, *BLBC* LBC with both parents migrating, *SLBC* LBC with one parent migrating, *NLBC* neither parents had migrated, *DC* children living in a divorced family without migrant parents

Adjusted by age, gender, income level, parental highest education level, sibling and household registration

*p < 0.05; **p < 0.01; ***p < 0.001; ^#^Dependent variable (suicidal ideation): 0 = I do not have any thoughts of killing myself

1 = I have thoughts of killing myself but I would not carry them out, I would like to kill myself, I would kill myself if I had the chance

^@^: n = 3883, adjusted R^2^ = 0.028, F = 9.85, p < 0.001; n = 3883, adjusted R^2^ = 0.192, F = 51.23, p < 0.001

^#^: n = 3878, adjusted R^2^ = 0.023, F = 8.28, p < 0.001; n = 3878, adjusted R^2^ = 0.275, F = 80.92, p < 0.001

^$^: n = 3913, Cox & Snell R^2^ = 0.009, Nagelkerke R^2^ = 0.016; n = 3913, Cox & Snell R^2^ = 0.060, Nagelkerke R^2^ = 0.109

^%^: n = 3909, Cox & Snell R^2^ = 0.041, Nagelkerke R^2^ = 0.060; n = 3909, Cox & Snell R^2^ = 0.142, Nagelkerke R^2^ = 0.208

Problems communicating with both fathers and mothers showed positive associations with total difficulties scores, internalizing problems scores and externalizing problems scores. Children who experienced higher levels of openness in mother-adolescent communication were less likely to have total difficulties (β = -0.06, p < 0.01) and externalizing problems (β = − 0.04, p < 0.01), and were more likely to exhibit pro-social behavior (β = 0.05, p < 0.01). Additionally, problems communicating with mothers, rather than with fathers, were strongly linked with NSSI (OR = 1.08; 95%CI = 1.05, 1.11; p < 0.001) and SI (OR = 1.09; 95%CI = 1.07, 1.12; p < 0.001). Overall, psychological resilience showed distinct patterns of associations with different subscale outcomes. Children who showed higher resilience scores tended to have lower total difficulties scores, lower internalizing problems scores and lower externalizing problems scores, and were more likely to have higher pro-social scores. In addition, children with higher levels of resilience reported being less likely to experience NSSI (OR = 0.99; 95%CI = 0.98, 0.99; p < 0.05) and SI (OR = 0.99; 95%CI = 0.98, 0.99; p < 0.001).

## Discussion

In the modern era, forms of childhood parental deprivation have become increasingly diversified. Dramatic economic growth and societal transformation in China have produced a large number of children whose parents have migrated to developed areas in search of better employment or whose parents have divorced. Gaining an understanding of the psychological adjustment of these children face is urgently needed. To the best of our knowledge, this is the first study to directly compare the mental health outcomes of children who experienced parental divorce or parental migration. We found that, after controlling for socio-demographic variables, DC were significantly more likely to have higher levels of internalizing problems, higher levels of externalizing problems, higher total difficulties score, and were less likely to show pro-social behaviors than were the other three groups. Furthermore, we observed a higher prevalence of NSSI and SI among DC when compared to the other three groups. We also observed significant associations between parent-adolescent communication and psychological resilience to the mental health of children.

The most important finding of this study is that the experience of prolonged separation from divorced parents had stronger negative effects on the mental health of children than was observed in left-behind children, after adjusting for socio-demographic factors. To explain the potential different impacts of various forms of parental separation, several possible explanations have to be considered. Parental migration affects the well-being of children through a trade-off between an increase in family income and a decrease in parental care. However, divorce often leads to economic disadvantage, which results in a lower socio-economic status (SES) in childhood [12]. As our study demonstrated, DC were about two times more likely than were the control group to come from poorer households. It is well understood that low childhood SES negatively affects psychological development and well-being [52, 53]. Although divorce has become very common and is much less stigmatized than it was in the past, divorce-related stigma still exists in much of rural China. This kind of stigmatization may create a high level of mental-emotional stress for children and thus increase their vulnerability to mental health problems [54]. However, LBC accounted for about 60% of all school children in the study area. Facing stigmatization from parental migration is relatively rare, and this finding is consistent with other research [55]. However, we were unable to examine the entirety of the family environment and its correlation with divorce because we lacked access to such information. It should be noted that marital conflict and negative interactions between parents, both before and after divorce, have been regarded as an important contributor to children’s problems [11]. Further study is necessary to more fully explore how family processes, such as multiple divorces, remarriage, and ongoing conflicts, mediate or moderate the adverse effects of divorce on children’s mental health outcomes.

After controlling for relevant socio-demographic variables, DC reported significantly higher rates of NSSI and SI. Consistent with previous studies, exposure to negative life events like parental divorce is a key factor associated with NSSI and SI [56]. In our study 18% of DC admitted to having hurt themselves deliberately during the past 12 months. Comparisons can be made with the recent nationwide survey in China on the adolescent health of grade 7–12 students, which reported that 12% of students had engaged in self-injury [26]. Additionally, previous self-injurious thoughts and behaviors are considered to be one of the strongest predictors of suicide attempts and suicide death [57]. Therefore, DC with suicidal ideation or non-suicidal self-injurious behaviors are at high risk for suicide. Targeted preventive strategies and socio-emotional support for these children should be prioritized in facilities such as schools or public health centers.

Interestingly, we found that children with divorced parents had similar SDQ and SITB outcomes to children with parents who migrated (either one or both parents migrating) when further adjusting for parent-adolescent communication and psychological resilience. The results of this study demonstrated worse mental health outcomes among DC than among BLBC and SLBC when adjustments were only made for socio-demographic confounders. However, after further adjusting for parent-adolescent communication and psychological resilience, DC no longer had higher levels of internalizing problems, externalizing problems, NSSI and SI. This suggests that parent-adolescent communication and psychological resilience are important protective factors for children’s mental health. From the family process perspective, separation is not necessarily as important to children’s later development as the quality of the parents’ relationship with their children [10]. As our study demonstrated, DC had the lowest scores on the openness sub-scales and total scales of father- and mother-adolescent communication, and had the highest scores on the problem subscales of father- and mother-adolescent communication, when compared with the other groups. As an indicator of the strength of the parent–child relationship, lack of parent-adolescent communication may result in huge challenges for the psychological development of children who are already deprived of parental care [21]. In addition, the forms and quality of caregiving children receive during parental absence due to divorce or migration are important and vary significantly, and future studies could investigate the relationship dynamics and childcare quality in various family and caregiving contexts.

Our study also suggests that low psychological resilience in children is related to mental health difficulties, NSSI and SI. Children with lower resilience are significantly more likely to experience all mental difficulties (internalizing problems, externalizing problems), self-injurious thoughts and behaviors. As has been reported in previous studies, the compensatory model of resilience underlines the role of protective factors in promoting psychological functioning and mitigating the negative influence of adversity [58]. Resilience-based intervention programs have been developed and implemented with various groups of vulnerable children and have proven their effects on fostering psychosocial well-being in low and middle-income countries [59]. A pilot trial developed in China also showed that resilience-based intervention programs are feasible and potentially efficacious in decreasing depression symptoms among migrant children [60]. Therefore, resilience-based intervention programs should be developed for children experiencing various forms of parental absence in order to best address this growing problem.

Our study had several limitations. First, the cross-sectional nature of our study prohibits causal inference. A longitudinal study is needed to explore the causal pathways and dynamic relationships between parental divorce and migration during childhood and in adult-onset disorders. Second, all analytical information we collected was self-reported, and therefore there exists the potential for recall bias. Further studies should collect data from multiple informants, such as residential parents or teachers, to present triangulation and achieve a more sophisticated data set. Third, our study only investigated a limited range of potential determinants, some variables, such as age at separation, length of separation from parents and parental history of mental disorders, were not measured in this study. Fourth, participants were sampled exclusively from two counties of Anhui province. Therefore, one should be caution when extrapolating and generalizing these results to children in China. We are still pursuing nationally representative data to gain a more complete understanding of the mental health and SITB of children in the context of massive parental separation.

## Conclusions

Despite the aforementioned limitations, our study underscores the risks associated with parental absence in childhood among Chinese children when their parents migrate to cities for employment or when their parents have divorced. As divorce rates increase and more residents migrate to large cities for work, the number of children left behind in their hometowns and living in divorced families will continue to rise. Gaining an understanding of the consequences of different forms of parental separation will provide us important context and knowledge for the development of interventions and prevention programs to promote these children’s well-being. In the last decades, the Chinese government has expressed concerns about the well-being, development, and human capital of LBC, and a number of policy announcements on these issues have been made. However, the government should also design special policy frameworks that provide support to DC, in light of the fact that children who suffer parental separation due to divorce are the most vulnerable group to mental health disorders. Additionally, the findings from this study contribute to existing knowledge by demonstrating that parent-adolescent communication and psychological resilience could serve as protective factors for children with benefits in mitigating the adverse effects of long-term parental absence because of parental divorce. It is recommended that further research be undertaken in developing and assessing the feasibility and efficacy of resilience-based or communication-based intervention programs for children in China.

## Data Availability

The datasets used during the current study are available from the corresponding author on reasonable request.

## Abbreviations

LBC: Left-behind children
BLBC: Left-behind children with both parents migrating
SLBC: Left-behind children with one parent migrating
N-LBC: Neither parents had migrated
DC: Children living in a divorced family without migrant parents
SITB: Self-injurious thoughts and behaviors
NSSI: Non-suicidal self-injury
SI: Suicidal ideation
PACS: Parent-adolescent communication scale
CD-RISC: Connor-Davidson resilience scale
SDQ: Strengths and Difficulties Questionnaire
